# Nerve Growth Factor Signaling Modulates the Expression of Glutaminase in Dorsal Root Ganglion Neurons during Peripheral Inflammation

**DOI:** 10.3390/ijms25116053

**Published:** 2024-05-31

**Authors:** Vikramsingh Gujar, Radhika D. Pande, Bhalchandra M. Hardas, Subhas Das

**Affiliations:** 1 Department of Anatomy and Cell Biology, Oklahoma State University, Center for Health Sciences, Tulsa, OK 74107, USA; 2 Department of Biochemistry and Microbiology, Oklahoma State University, Center for Health Sciences, Tulsa, OK 74107, USA; radhika.pande@okstate.edu (R.D.P.); subhas.das@okstate.edu (S.D.); 3 Department of Electronics Engineering, Shri Ramdeobaba College of Engineering and Management, Nagpur 440013, India; hardasbm@rknec.edu

**Keywords:** inflammation, nerve growth factor (NGF), peripheral inflammation, nociception, NGF-TrkA signaling, dorsal root ganglion, glutaminase, Rab7, Rab7GTPase

## Abstract

Glutamate functions as the major excitatory neurotransmitter for primary sensory neurons and has a crucial role in sensitizing peripheral nociceptor terminals producing sensitization. Glutaminase (GLS) is the synthetic enzyme that converts glutamine to glutamate. GLS-immunoreactivity (-ir) and enzyme activity are elevated in dorsal root ganglion (DRG) neuronal cell bodies during chronic peripheral inflammation, but the mechanism for this GLS elevation is yet to be fully characterized. It has been well established that, after nerve growth factor (NGF) binds to its high-affinity receptor tropomyosin receptor kinase A (TrkA), a retrograde signaling endosome is formed. This endosome contains the late endosomal marker Rab7GTPase and is retrogradely transported via axons to the cell soma located in the DRG. This complex is responsible for regulating the transcription of several critical nociceptive genes. Here, we show that this retrograde NGF signaling mediates the expression of GLS in DRG neurons during the process of peripheral inflammation. We disrupted the normal NGF/TrkA signaling in adjuvant-induced arthritic (AIA) Sprague Dawley rats by the pharmacological inhibition of TrkA or blockade of Rab7GTPase, which significantly attenuated the expression of GLS in DRG cell bodies. The results indicate that NGF/TrkA signaling is crucial for the production of glutamate and has a vital role in the development of neurogenic inflammation. In addition, our pain behavioral data suggest that Rab7GTPase can be a potential target for attenuating peripheral inflammatory pain.

## 1. Introduction

The involvement of the nerve growth factor (NGF) is well documented in the development of the peripheral nervous system and in determining the phenotype of sensory and sympathetic neurons. In addition, NGF levels increase during various inflammatory conditions and are involved in inducing pain and hyperalgesia [1,2,3]. NGF can bind to its high-affinity receptor, tropomyosin receptor kinase A (TrkA), and its low affinity, p75 neurotrophin receptor (p75NTR). The NGF secreted by resident and inflammatory cells and keratinocytes in injured tissue acts via TrkA receptors present on primary sensory nerve endings and participates in peripheral sensitization [4]. Hence, NGF/TrkA signaling is considered as a primary target for the development of pain therapeutics [5] leading to the development of anti-NGF antibodies and TrkA antagonists [6,7]. However, for these analgesics to be effective with the fewest possible side effects, the detailed mechanisms involved in peripheral sensitization need to be evaluated further.

For blocking NGF signaling, several drugs have been developed, such as NGF protein inhibitors (Anti-NGF antibodies), NGF/TrkA binding inhibitors, and TrkA inhibitors [8,9,10,11]. TrkA is present on the cell membranes of various cell types, including primary sensory neurons (intraepidermal nerve fibers), immune, and skin cells. After the binding of NGF to TrkA, the complex undergoes dimerization and autophosphorylation on the axonal terminals of primary sensory neurons, followed by endocytosis either in a clathrin-dependent or an independent manner. The signaling endosome is retrogradely transported to the cell bodies in the dorsal root ganglion (DRG). Before the long-range transport is initiated, the TrkA-positive endosomes accumulate the small GTPase from the Rab family, i.e., Rab7GTPase in the peripheral terminal [12]. Rab GTPases are the molecules that determine whether the endosome should travel retrogradely to the cell body for signal transduction or degradation in lysosomes. Once the TrkA-signaling endosome associates with Rab7, it moves retrogradely and initiates the downstream signaling for the expression of different inflammatory mediators and neurotransmitters [13]. Therefore, in this study, we used TrkA and Rab7 pharmacological inhibitors to block the NGF signaling during adjuvant-induced arthritis (AIA) to determine the effect of NGF on glutaminase (GLS) levels in DRG neurons and pain behavior in AIA animals.

All DRG neurons are glutamatergic as they utilize glutamate to convey information from their peripheral and central terminals, with glutamate, the most abundant amino acid in the human body, which shows unique structuring and bioactivity even as an isolated peptide [14]. Previous studies linked the peripheral release of glutamate with the process of nociception and sensitization of primary afferent DRG neurons [15]. Levels of glutamate are elevated in the synovial fluid and epidermis of patients with arthritis and gold-induced skin inflammation, respectively [16,17]. The primary afferents convert glutamine to glutamate via the enzyme GLS. This GLS is activated regionally by calcium and phosphate and, hence, also known as phosphate-activated glutaminase (PAG) [18]. We previously reported that the level of GLS is elevated significantly in DRG cell bodies during chronic inflammation, and peripheral inhibition of GLS reduces sensitization and pain associated with inflammation [15,19,20,21].

After the initiation of the inflammatory process, the DRG sensory neurons alter the expression of proteins such as Substance P (SP) and Calcitonin gene-related peptide (CGRP), which is mostly attributed to NGF and its high-affinity receptor, TrkA. Although, under normal conditions, the basal expression of GLS is not regulated by NGF [22] during the process of inflammation, the role of NGF/TrkA in the regulation of GLS expression needs further evaluation. Determining if NGF/TrkA signaling directly affects GLS levels in DRG neurons and evaluating Rab7-mediated retrograde signaling as a therapeutic target for pain management is necessary. The goal of this study was to explore the involvement of NGF signaling in the process of neurogenic inflammation by evaluating the levels of GLS in DRG neurons by inhibiting TrkA and Rab7 during peripheral inflammation. The hypothesis is that selective TrkA inhibition in the periphery will block the formation of NGF/TrkA complex, while the inhibition of Rab7 will disrupt the NGF/TrkA long-range retrograde signaling, hence affecting the alteration in DRG cell bodies. We used GW441756 (TrkA inhibitor) and CD1067700 (Rab7 inhibitor) to block the NGF signaling in the periphery and evaluated the expression of GLS in DRG cell bodies. We observed that inhibition of both TrkA and Rab7 individually attenuated the levels of GLS, thereby confirming the involvement of NGF retrograde signaling in the glutamate metabolism. We also established the potential of Rab7 as a therapeutic target for treating pain, as Rab7 blockade showed reduced pain behavior in arthritic animals.

## 2. Results

### 2.1. Effect of AIA on Levels of NGF, pTrkA, and Rab7 in Sciatic Nerve

**Figure 1:**
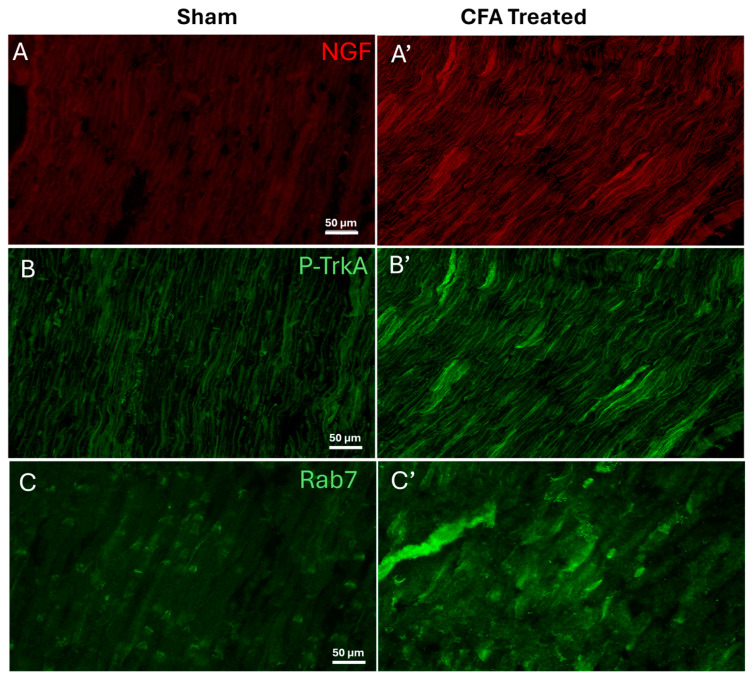
Peripheral inflammation elevates the levels of NGF, pTrkA, and Rab7GTPase in ligated sciatic nerve. Increased levels of NGF (**A**,**A’**), pTrkA (**B**,**B’**), and Rab7 (**C**,**C’**) were detected in sciatic nerve of CFA-treated animals as compared to sham animals, as determined by qualitative immunohistochemistry. Scale bar = 50 μm, (*n* = 3).

For determining the levels of proteins associated with NGF signaling in the sciatic nerve during peripheral inflammation, we ligated the sciatic nerve of adjuvant-induced arthritic animals. The ligation obstructed the axonal transport, allowing us to evaluate the levels of NGF, pTrkA, and Rab7. Representative images of the ipsilateral sciatic nerve showed a qualitative increase in the immunoreactivity of NGF, pTrkA, and Rab7 (Figure 1).

**Figure 2:**
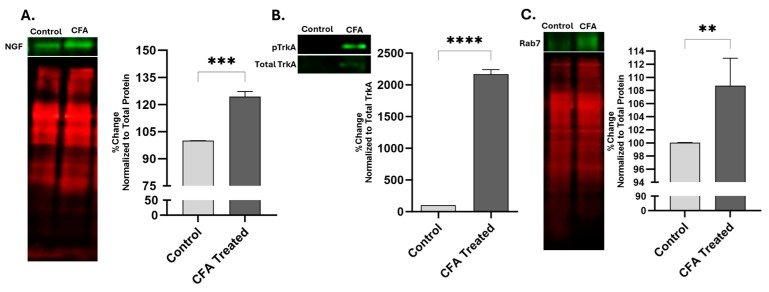
Peripheral inflammation elevates the levels of NGF, pTrkA, and Rab7GTPase in sciatic nerve after ligation. Increased levels of NGF (**A**), pTrkA (**B**), and Rab7 (**C**) were detected in sciatic nerve of CFA-treated animals as compared to control rats after 48 h of inflammation, as determined by fluorescence western blot. The NGF and Rab7 data were normalized by total protein, while pTrkA data was normalized against total TrkA. ** *p* < 0.01, *** *p* < 0.001, **** *p* < 0.0001. (*n* = 4).

Western blot data detected the band for the pro-NGF form at 27 KDa, confirming previous studies [23]. The NGF-ir was elevated in the sciatic nerve of AIA animals after the initiation of inflammation (Figure 2A). After 48 h of AIA, the levels of pTrkA, compared to the total TrkA, were found to be significantly higher (** *p* = 0.0061, 95% CI—1364 to 2760) in the sciatic nerve with ligation (Figure 2B). Quantitative western data indicated a significant increase in the immunoreactivity of Rab7 in the sciatic nerve of CFA-treated animals compared to the sham animals (Figure 2C).

### 2.2. Effect of TrkA Inhibition on GLS Expression during AIA

**Figure 3:**
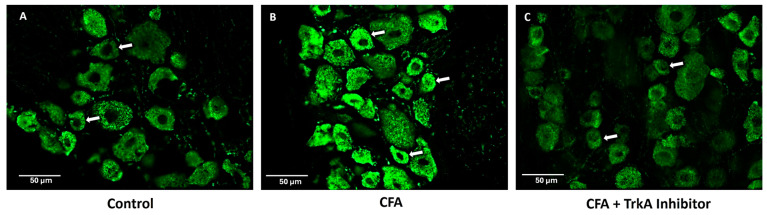
Selective TrkA inhibition attenuates GLS levels in DRG during peripheral inflammation: Representative images showing GLS immunoreactivity (green). (**A**) Expression of GLS in DRG of naïve animal. (**B**) GLS expression in DRG of AIA animal after 48 h of inflammation. (**C**) GLS expression in animal treated with CFA and selective TrkA inhibitor (GW441756). Note the expression of GLS in small diameter neurons (white arrows) in animals treated with CFA is higher as compared to the animals treated with TrkA inhibitor along with CFA. Scale bar = 50 μm was applied to all photomicrographs. (*n* = 3).

The levels of GLS were evaluated after 48 h in the L4 and L5 DRGs of rats treated with CFA and selective TrkA inhibitor (GW441756). Almost all the DRG neurons were immunoreactive for GLS, as reported in the previous studies [15,21,24,25]. The GLS-ir was elevated in the animals treated with CFA as compared to naïve rats. TrkA pharmacological inhibition with selective TrkA inhibitor attenuated the GLS-ir in the DRG neurons compared to the neurons of CFA-treated animals (Figure 3).

**Figure 4:**
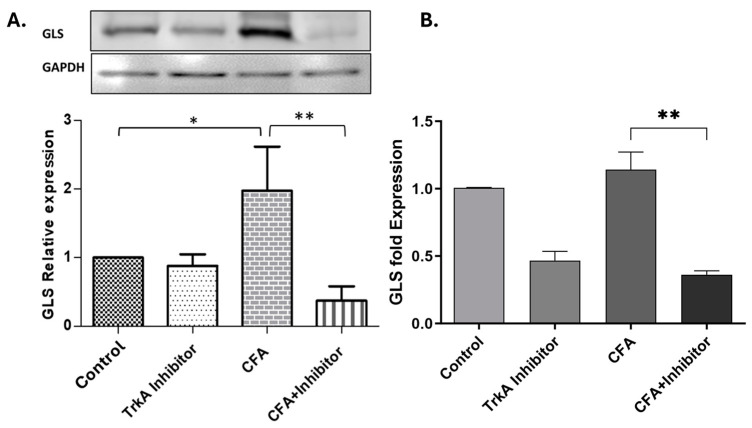
Selective inhibition of TrkA in the periphery during AIA changes the expression of GLS expression in rat DRG neurons after 48 h of inflammation. (**A**) The levels of GLS protein are upregulated in animals treated with CFA as compared to control but decreased significantly in the group treated with TrkA inhibitor and CFA, as determined by quantitative western blot analysis. (**B**) The GLS mRNA analysis showed that peripheral TrkA inhibition decreased GLS mRNA in both control and CFA-treated rats, as determined by qRT-PCR. * *p* < 0.05, ** *p* < 0.01 (Student’s *t*-test; *n* = 3).

The western blot data indicated a significant reduction in the levels of GLS in the DRG cell bodies of animals treated with TrkA inhibitor compared to AIA animals (Figure 4A). We also evaluated the levels of GLS mRNA in DRGs to determine the transcriptional changes due to the NGF signaling blockade via TrkA inhibition. The GLS mRNA levels of TrkA-inhibited rats were significantly diminished compared to the CFA-alone group (Figure 4B).

### 2.3. Effect of Rab7 Inhibition on GLS Expression and Pain Behavior during AIA

**Figure 5:**
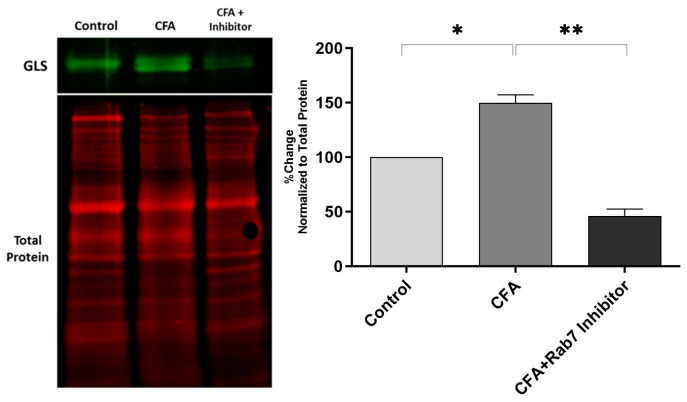
Inhibition of Rab7 in the periphery during AIA, changes the expression of GLS in rat DRG neurons after 48 h of inflammation. Image showing GLS bands (66 and 68 kDa) and total proteins after western blotting. The expression of GLS is significantly reduced in the group CFA + Inhibitor as compared to CFA after normalizing with total protein (95% CI −146.7 to −60.98). * *p* < 0.05, ** *p* < 0.01. (*n* = 4).

The peripheral inhibition of Rab7GTPase during CFA-induced inflammation decreased the GLS-ir in the L4 and L5 DRGs. The quantitative western blot data show the elevation of GLS after peripheral inflammation, confirming previous findings [19,21,24,26]. After the Rab7 inhibition, the GLS levels were attenuated significantly in the DRGs of AIA rats (Figure 5).

**Figure 6:**
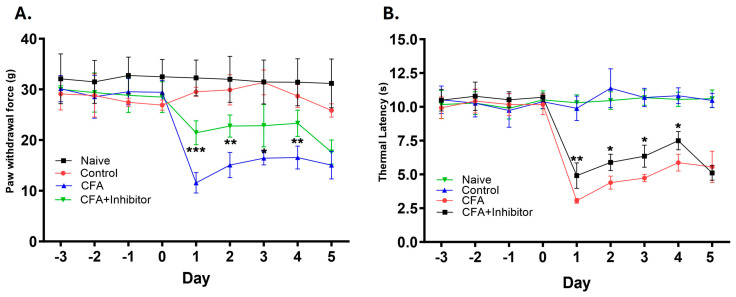
Mechanical thresholds and thermal latencies from rat hind paws after inhibition of Rab7 in the periphery during AIA. (**A**) Inhibition of Rab7 in the periphery during AIA (CFA + Inhibitor), caused a significant increase in paw withdrawal force from Days 1 through 4 as compared to the CFA group. (**B**) Inhibition of Rab7 in the periphery during AIA (CFA + Inhibitor) caused significant increase in the thermal latencies from Days 1 through 4 as compared to the CFA group. Data are graphed as means (±SD). * *p* < 0.05, ** *p* < 0.01, *** *p* < 0.001. (Student’s *t*-test; *n* = 5).

Pain behavior was determined by measuring the mechanical thresholds and thermal latencies in the hind paws of animals after injecting and topically applying the CFA and Rab7 receptor antagonist. The reduction in paw withdrawal force and thermal withdrawal latency was interpreted as the presence of mechanical and thermal hyperalgesia, respectively. The mechanical threshold values for animals treated with CFA were significantly reduced compared to control animals starting at 24 h and remained reduced until Day 5 (*p* ≤ 0.001). The treatment with Rab7 inhibitor along with CFA (Group: CFA + Inhibitor) significantly reduced the mechanical hyperalgesia as compared to the CFA-only group after Day 1 (*p* < 0.001), Day 2 (*p* < 0.01), Day 3 (*p* < 0.05), and Day 4 (*p* < 0.01) (Figure 6A). Similarly, the thermal withdrawal latencies were increased significantly in animals treated with CFA and inhibitor as compared to the animals treated with only CFA. The reduced thermal hyperalgesia was observed after Day 1 (*p* < 0.01), Day 2 (*p* < 0.05), Day 3 (*p* < 0.05), and Day 4 (*p* < 0.05) in rats treated with the Rab7 receptor antagonist with CFA (Figure 6B).

## 3. Discussion

### 3.1. Levels of NGF, pTrkA, and Rab7 Increases during AIA in Sciatic Nerve

Our study showed that the unilateral injection of CFA in the rats’ hind paws elevates the levels of NGF, pTrkA, and Rab7 in the sciatic nerve. These results confirmed the previous findings of the involvement of the NGF/TrkA axis in the development of peripheral inflammation [3,4,22,23,27,28]. The levels of NGF are elevated in the cerebrospinal fluid and synovial fluid of patients suffering from inflammatory and autoimmune diseases such as multiple sclerosis and rheumatoid arthritis, respectively [29,30]. Different animal models of induced arthritis show increased NGF expression [2,31]. Neuropeptides like SP and CGRP, and cytokines like IL-6 and IL-β, are released after the noxious stimuli and are primary mediators of regulating the inflammatory process. This release of inflammatory mediators might elevate the NGF basal levels, as predicted previously [28,32].

The NGF antibody we used in this study can bind to mature (13 kDa) and pro-NGF (27 and 35 kDa) forms [33] simultaneously; hence, we cannot differentiate between the two forms in the immunohistochemistry data. Our western blot analysis also failed to detect the mature NGF (β-NGF), while the pro-NGF form gave a prominent band at 27 kDa. The method of tissue processing and western blotting we employed in this study might be the reason for not detecting mature NGF. Issues like protein degradation, inefficient extraction, and non-specific antibody binding can negatively impact detection. To improve future studies, we suggest several alternatives: rapid sample handling and optimized extraction buffers to prevent protein degradation; using high-affinity antibodies, better blocking and washing techniques, and optimized transfer conditions in western blotting; and considering other detection methods such as ELISA for better sensitivity, immunohistochemistry for spatial information, mass spectrometry for high specificity, and quantitative PCR to complement protein detection.

TrkA is the high-affinity receptor for NGF and is expressed in various types of cells, including peripheral sensory neurons. After binding to NGF on the cell membrane, TrkA is activated and phosphorylates the tyrosine residues present on the cytoplasmic domain, thus converting from TrkA to pTrkA [34,35]. Therefore, for determining the expression of TrkA during the process of inflammation, we used the pTrkA antibody and normalized its expression with total TrkA. After the initiation of inflammation and disrupting the NGF trafficking by sciatic nerve ligation, we found that the levels of pTrkA compared to total TrkA were significantly increased (*p* = 0.0061) in the sciatic nerve. Previous studies demonstrated that the inflammatory stimulation significantly elevates TrkA expression in immunologic cells like monocytes, macrophages, B lymphocytes, and T lymphocytes [36], therefore some of the TrkA inhibition may occur among these cells, also. In addition, the TrkA immunoreactivity and phosphorylation are shown to be strongly upregulated in the lumbosacral DRGs of animals with cyclophosphamide (CYP)-induced cystitis [37], but we did not attempt to detect these in the DRG in the current study.

We also found the levels of Rab7GTPase increased in the sciatic nerve along with NGF and pTrkA. The Rab family of GTPase is crucial for organelle transport and is responsible for regulating different processes involved in membrane trafficking pathways [12]. The endosomal GTPase Rab7 is functionally distinct from other members of the Rab superfamily and has been implicated in the transport of late endosomes [38]. After the endocytosis of the NGF/TrkA complex, the signaling endosome travels retrogradely to the cell bodies to initiate signal transduction. Before the initiation of retrograde transport, the TrkA-positive endosomes undergo maturation, forming Rab5GTPase-positive early endosomes to Rab7GTPase-positive late endosomes [39]. Furthermore, a mutation in Rab7 leads to defective axonal transport and dysregulated NGF/TrkA signaling in DRG neurons, suggesting Rab7’s pivotal role in the trafficking of TrkA-positive endosomes [40].

### 3.2. Selective TrkA Inhibition Attenuates GLS Expression during Peripheral Inflammation

GLS plays a crucial role in the glutamine–glutamate cycle. Several studies documented the immunoreactivity of GLS in all sizes of DRG neurons [21,24]. Our lab previously showed that GLS-immunoreactivity (-ir) and enzyme activity are elevated in DRG neuronal cell bodies during chronic peripheral inflammation [21], but the mechanism for this GLS elevation has not been fully characterized. In this study, we found that the NGF signaling blockade in the periphery, via selective TrkA pharmacological inhibition, attenuated the levels of GLS in the DRG during AIA, confirming the interplay between NGF signaling and glutamate metabolism [22]. During the inflammatory process, elevated amounts of GLS are anterogradely transported to the peripheral nerve terminals, causing an increase in glutamate production. The elevated glutamate production and release is responsible for sensitizing the primary sensory afferents, thereby regulating nociceptive transmission [26]. The data from this study implicate that the NGF signaling inhibition eventually attenuates the glutamate production in the periphery, leading to maintenance in nociceptive sensitization.

TrkA inhibition indicates other potential ways of inhibiting NGF signaling apart from anti-NGF antibodies. NGF has a high affinity for TrkA receptors and a low affinity for p75 neurotrophin receptors present at the axonal terminal. Binding of NGF to TrkA promotes endocytosis and the formation of the signaling endosomes. These NGF/TrkA signaling endosomes are structurally and molecularly defined as multivesicular bodies. For eliciting the transcriptional modulation in the cell soma, NGF/TrkA endosomes undergo long-range retrograde travel from the distal axons to the DRG cell bodies for initiating subsequent downstream intracellular signaling [41,42,43]. TrkA activation elevates the expression of SP and CGRP, leading to the sensitization of nociceptors during inflammation [44,45]. Therefore, selective TrkA inhibition can decrease inflammation and sensitization, thus reducing the pain behavior in conditions with a prominent inflammatory process like arthritis [46,47]. In addition, pharmacological inhibition of TrkA can also be used for studying the mechanisms involved in the trafficking of NGF signaling endosomes during the process of nociception.

### 3.3. Peripheral Inhibition of Rab7GTPase Decreases the GLS Expression and Provides Analgesia

Our study shows the pharmacological inhibition of small GTPase Rab7 via a receptor antagonist in the periphery attenuates the GLS levels in DRG cell bodies of primary sensory neurons after the initiation of inflammation. Previous studies have linked the Rab7 with the TrkA-positive endosomes in neurons and retrograde transport from the axon to the neuronal cell body [40,48]. This makes Rab7 a potential target for disrupting the NGF signaling. The primary function of the Rab7 is to regulate the maturation of early endosomes into late endosomes, and it is associated with fusion and clustering [49]. As Rab7 interacts with TrkA in the multivesicular bodies, Rab7 inhibition causes an excess of TrkA to be accumulated in the endosomes and hindrance of TrkA endosomal trafficking [12]. We postulate that the inhibition of NGF/TrkA signaling via Rab7 is responsible for modulating the levels of GLS in the DRG, hence suggesting the potential mechanism behind the regulation of glutamate metabolism during peripheral inflammation.

The findings of this study also provide evidence for the analgesic potential of Rab7 inhibition, as the Rab7 receptor antagonist CD1067700 reduced pain behavior associated with the adjuvant-induced inflammation. This offers a novel therapeutic strategy for alleviating arthritic pain. We found the mechanical and thermal hyperalgesia were reduced significantly after Day 1 in groups treated with Rab7 inhibitor. As the long-range NGF/TrkA retrograde signaling takes a considerable amount of time to reach the DRG cell bodies and exert transcriptional changes, the early analgesic effect might be due to the interaction of Rab7 with various other pain-related molecules, such as μ-opioid receptors and calcium-/calmodulin-dependent protein kinase 4. These studies suggest Rab7’s interaction with different molecules related to pain processing might be necessary for the development of nociceptive processes [50]. In addition, the involvement of Rab7 in the hereditary sensory neuropathies (HSN) and an autosomal dominant inherited disorder causing peripheral neuropathy, Charcot-Marie-Tooth (CMT), provides a rationale to study this small GTPase closely.

## 4. Materials and Methods

### 4.1. Animals

Sprague Dawley rats (350–450 g, N = 99) bred and housed in the OSU-CHS animal facility were used in this study. Animals were maintained on a 12 h light:12 h dark cycle and provided with continuous access to food and water. These studies were performed at Oklahoma State University-Center for Health Sciences (OSU-CHS), and the procedures are approved by the OSU-CHS Institutional Animal Care and Use Committee. All the procedures were performed according to the National Institute of Health and International Association for the Study of Pain guidelines [51].

### 4.2. Induction of Adjuvant-Induced Arthritis (AIA) and Sciatic Nerve Ligation

For inducing unilateral inflammation of the hind paw, Complete Freund’s Adjuvant (CFA; Sigma; St. Louis, MO, USA) was used. Rats (*n* = 12) were anesthetized with isoflurane (initially 5%, then reduced to 2.5%), and 150 µL of a 1:1 emulsion containing CFA and sterile phosphate-buffered saline (PBS) was injected into the plantar surface of the right hind paw. Rats were allowed to recover on a warm towel and then placed back in their cages. A total of 6 h after CFA treatment, animals were anesthetized again, and the right sciatic nerve was exposed and ligated with non-absorbable silk before the trifurcation, 5 mm below the sciatic notch [52]. Sham animals were used as a control group in which the same surgery was performed, but no CFA injection was administered.

### 4.3. Adjuvant-Induced Arthritis (AIA) and Pharmacological Interventions

Rats were anesthetized prior to all injections, and AIA was induced by injecting 150 µL of CFA into the plantar surface of the right hind paw. TrkA inhibitor GW441756 (Tocris Biosciences, Bristol, UK) and Rab7 receptor antagonist CD1067700 (Cayman, Ann Arbor, MI, USA) were dissolved in 5% DMSO in PBS and injected as 20 nmol/20 µL and 100 nmol/20 µL, respectively, in the plantar surface of the right hind paw. Besides the injection, the inhibitors were also applied topically onto the skin with the help of filter paper soaked in DMSO and inhibitor. The inhibitors were injected and topically applied once before 30 min of CFA treatment and two times, 12 h and 36 h, after CFA treatment. Rats with respective-volume PBS injections as compared to the treated rats were used as controls [53].

### 4.4. Immunofluorescence

The animals (*n* = 9) were perfusion-fixed with a fixative containing 0.96% (*w*/*v*) picric acid and 0.2% (*w*/*v*) formaldehyde in 0.1 M sodium phosphate buffer, pH 7.3 [22,54]. The sciatic nerve and the L4 and L5 DRGs were collected from the perfusion-fixed rats and immersed in the same fixative for 4 h at room temperature. The tissues were transferred to a 10% sucrose solution in PBS (pH 7.3) and incubated at 4 °C overnight. The next day, the tissues were embedded and frozen in Lipshaw embedding matrix and cut in 14 μm sections on a Leica CM 1850 cryostat (Leica Biosystems, Wetzlar, Germany). The slices were collected on gelatin-coated slides and dried on a slide warmer at 37 °C for 1 h. After rinsing the slides with 1X PBS, the slides were incubated with the primary antibodies mentioned in Table 1 for 4 days at 4 °C [54]. The slides were washed three times with 1X PBS, incubated with respective fluorescent-labeled secondary antibodies (Table 1) for 60 min at room temperature in the dark. Finally, after incubation with secondary antibodies, the slides were rinsed thrice with 1X PBS and coverslipped with ProLong Gold Mounting Media (Invitrogen; Carlsbad, CA, USA) for image analysis.

### 4.5. Western Blot Analysis

The sciatic nerve and L4, L5 DRGs were homogenized in lysis buffer (25 mM Tris HCl pH-7.4, 150 mM NaCl, 151 1 mM EDTA, 5% glycerol, and 1% Triton X-100) added with a protease inhibitor cocktail (Sigma-Aldrich, St. Louis, MO, USA) for 5 min with a handheld homogenizer and incubated on ice for 10 min. The supernatant was collected in a fresh tube after centrifugation of the homogenized sample at 14,000 rpm for 15 min at 4 °C. Total protein was estimated by Pierce™ BCA Protein Assay Kit (Thermo Fisher Scientific, Waltham, MA, USA). The samples (20–50 µg/mL of total protein) were dissolved in loading buffer (10 mM Tris Base, 1 mM EDTA, 2.5% SDS, 5% β-mercaptoethanol, and 0.01% bromophenol blue) and boiled at 100 °C for 10 min. The sciatic nerve protein samples were separated on 12% Gel, and DRG samples were separated on 7.5% Gel (TGX™ FastCast™ Acrylamide Solutions, Bio-Rad Laboratories, Hercules, CA, USA) along with Spectra™ Multicolor Broad Range Protein Ladder (Thermo Fisher Scientific, Waltham, MA, USA) by SDS-PAGE and blotted onto nitrocellulose membrane in Mini Trans-Blot Cell (Bio-Rad Laboratories, Hercules, CA, USA).

TrkA inhibition study: The membranes were incubated with 5% Carnation milk in the Tris-buffered saline Tween (TBST, 20 mM Tris-HCl, 150 mM NaCl, 0.05% Tween 20, pH 7.5) at room temperature for 1 h and incubated overnight at 4 °C in the primary antibody against GLS (Table 2). After washing with 1XTBST thrice (20 min each), the membranes were incubated in alkaline phosphatase-labeled anti-rabbit IgG (Promega; Madison, WI, USA) secondary antibodies at 1:1000 dilution for 120 min. After washing with 1XTBST three times for 20 min each, the ECF substrate was used on a Typhoon 9410 Variable Mode Imager for taking the western blot images. The images were analyzed by ImageJ (Version—1.53t, National Institute of Health, Bethesda, MD, USA).

Sciatic nerve ligation and Rab7 inhibition study: After the electrophoretic transfer, the membranes were rinsed with Tris-buffered saline (TBS, 20 mM Tris-HCl, 150 mM NaCl, 0.05%, pH 7.5), incubated in Revert Total Protein Stain (Li-Cor Biosciences, Lincoln, NE, USA) for 5 min, and rinsed with Revert wash solution and blocked with 5% Carnation milk in TBS at room temperature for 1 h. All membranes were incubated overnight at 4 °C with the primary antibodies against NGF, pTrkA, Rab7, and GLS (Table 2) diluted in TBST (20 mM Tris-HCl, 150 mM NaCl, 0.05% Tween 20, pH 7.5). After washing with TBST four times (5 min each), the membranes were incubated for 1 h at room temperature in IRDye 800- and IRDye 680-labeled Donkey anti-rabbit and anti-mouse secondary antibodies (1:20,000). The membranes were rinsed with TBS (no Tween), followed by image acquisition on the Odyssey CLx Infrared Imaging system (Li-Cor Biosciences, Lincoln, NE, USA). The images were normalized against the total protein or total TrkA, and analysis was performed on Image Studio Lite software (Version 3.1, Li-Cor Biosciences, Lincoln, NE, USA).

### 4.6. Quantitative Reverse Transcription PCR

DRGs were collected in liquid nitrogen and stored at −80 °C until further use. The total RNA was extracted using a Trizol solution (Thermo Fisher Scientific, Waltham, MA, USA). The RNA was reverse-transcribed using M-MLV reverse transcriptase (Promega, Madison, MI, USA), and quantitative real-time PCR (qRT-PCR) was performed using the ABI StepOneTM system (Applied Biosystems, Foster City, CA, USA). GAPDH was used as an internal reference with a primer sequence (Table 3). The data were expressed as 2^−ΔΔCt^, which represents the relative amount of target mRNA present in the treated sample to the naïve animal group.

### 4.7. Pain Behavior

The animals (n = 45) were tested for pain behavior by determining the mechanical thresholds and thermal latencies after the initiation of inflammation with CFA. Before the testing started, the rats were familiarized in the behavioral room by handling them for at least 3 days (twice every day for 1 h). The baseline values were recorded for 3 days (Days −3, −2, −1) before the initiation of inflammation. On the day of treatment (Day 0), the values were recorded before the CFA injection. All behavioral testing was performed at the same time (late afternoon, around 3 PM) every day to avoid any variation in animal responses. A total of nine readings were obtained, including four at baseline testing (Days −3, −2, −1 and 0) and five after CFA treatment (Days 1, 2, 3, 4, and 5). Mechanical threshold values were obtained using a Dynamic Plantar Aesthesiometer (Ugo Basile, Gemonio, Italy). The maximum force was set to 50 g with the ramp rate of 5 g/sec. Thermal latencies were measured using a Plantar Test apparatus (Ugo Basile, Gemonio, Italy) with the maximum cut-off value set at 55 °C and the intensity set at 55 mW/cm^2^. All the tests were performed by placing the animal in the acrylic apparatus, and readings were obtained once the exploratory behavior ceased. For both mechanical threshold and thermal latency, five readings at intervals of 5 min were recorded from the ipsilateral hind paw of each rat.

The PCR analysis results were reported as the threshold cycle (Ct), which determined the mRNA of the target gene in relation to the reference gene. The difference between the number of cycles required to detect the PCR products for the target and reference genes was represented by ΔCt. ΔΔCt was the difference between the naïve animal group and the AIA group. Finally, the relative amount of target mRNA in the CFA-treated sample compared to the control animal group was expressed as 2^−ΔΔCt^.

### 4.8. Statistical Analysis

All the data were subjected to Student *t*-tests using GraphPad Prism (version 5.01 for Windows, GraphPad Software, San Diego, CA, USA). *p* values less than 0.05 were considered significant for all tests. The data presented in the graph are grouped by mean ± SEM (and/or SD), and the 95% CI is reported.

## 5. Conclusions

In the adjuvant-induced animal model of persistent inflammation, we have demonstrated that the peripheral inhibition of TrkA and Rab7 can modulate the expression of GLS in the DRG, thus affecting the primary afferent’s sensitization process. Our data suggest the Rab7GTPase inhibitor CD1067700 provides an analgesic effect in arthritic animals via the NGF signaling blockade. Further studies directed towards Rab7 are necessary to understand the trafficking of the NGF signaling endosome during the process of inflammation and nociception. We predict that such information on the NGF/TrkA axis can provide potential therapeutic targets for treating chronic inflammation.

## Figures and Tables

**Table 1:** Details of primary and secondary antibodies used in immunofluorescence studies.

Tissue	Primary Antibodies	Dilutions	Secondary Antibodies	Dilutions
Sciatic Nerve	NGF Anti-mouse (E-12, Santa Cruz, TX, USA)	1:1000	Donkey anti-mouse Alexa Flour 555 (Invitrogen, Carlsbad, CA, USA)	1:1000
	pTrkA Anti-rabbit (4168S, Cell Signaling, Danvers, MA, USA)	1:2000	Donkey anti-rabbit FITC 488 (Invitrogen, Carlsbad, CA, USA)	1:1000
	Rab7 Anti-rabbit (55469-1-AP, Proteintech, Rosemont, IL, USA)	1:2000	Donkey anti-rabbit FITC 488 (Invitrogen, Carlsbad, CA, USA)	1:1000
L4 and L5 DRG	GLS Anti-rabbit (Norman Curthoys, Colorado State University, Fort Collins, CO, USA)	1:1000	Donkey anti-rabbit FITC 488 (Invitrogen, Carlsbad, CA, USA)	1:1000

**Table 2:** Details of primary and secondary antibodies used in western blot studies.

Study/Tissue	Primary Antibodies	Dilutions	Secondary Antibodies	Dilutions
Sciatic Nerve Ligation/Sciatic Nerve	NGF Anti-Mouse (E-12, Santa Cruz, TX, USA)	1:1000	IRDye 800CW Donkey anti-mouse (Li-Cor, Lincoln, NE, USA)	1:20,000
	pTrkA Anti-rabbit (4168S, Cell Signaling, Danvers, MA, USA)	1:2000	IRDye 800CW Donkey anti-rabbit (Li-Cor, Lincoln, NE, USA)	1:20,000
	Rab7 Anti-rabbit (55469-1-AP, Proteintech, Rosemont, IL, USA)	1:2000	IRDye 800CW Donkey anti-rabbit (Li-Cor, Lincoln, NE, USA)	1:20,000
	Total TrkA Anti-rabbit (2505S, Cell Signaling, Danvers, MA, USA)	1:2000	IRDye 800CW Donkey anti-rabbit (Li-Cor, Lincoln, NE, USA)	1:20,000
TrkA Inhibition/L4, L5 DRG	GLS Anti-rabbit (Norman Curthoys, Colorado State University, Ft. Collins, CO, USA)	1:1000	AP labeled Anti-rabbit IgG (Promega, Madison, WI, USA)	1:1000
Rab7 Inhibition/L4, L5 DRG	GLS Anti-rabbit (Norman Curthoys, Colorado State University, Ft. Collins, CO, USA)	1:1000	IRDye 800CW Donkey anti-rabbit (Li-Cor, Lincoln, NE, USA)	1:20,000

**Table 3:** Primer sequences used for quantitative PCR.

Gene	Primer Sequence
GLS	GLS-F: 5′-GGGTCTGTTACCTAGCTTGGAAGATTTGC-3′ GLS-R: 5′-GAGTTAATCTTAACATATCCATACACT-3′
GAPDH	GAPDH-F: 5′-GAACCACGAGAAATATGACAACTCCCTCAAG-3′ GAPDH-R: 5′-GCAGTGATGGCATGGACTGTGG-3′

## Data Availability

All relevant data are included in the manuscript.
